# Feasibility evaluation of micro-optical coherence tomography (μOCT) for rapid brain tumor type and grade discriminations: μOCT images versus pathology

**DOI:** 10.1186/s12880-019-0405-6

**Published:** 2019-12-30

**Authors:** Xiaojun Yu, Chi Hu, Wenfei Zhang, Jie Zhou, Qianshan Ding, M. T. Sadiq, Zeming Fan, Zhaohui Yuan, Linbo Liu

**Affiliations:** 1 School Automation, Northwestern Polytechnical University, Xi’an, Shaanxi 710072 People’s Republic of China; 2 School of Automation, Northwestern Polytechnical University, NO. 1 Dongxiang Road, Chang’an District, Xi’an, Shaanxi 710129 People’s Republic of China; 3 Department of Neurosurgery, Renmin Hospital, Wuhan University, Wuhan, 430060 People’s Republic of China; 4 School of Medicine, Northwest University, Xi’an, 710069 People’s Republic of China; 5 School of Electrical & Electronic Engineering, Nanyang Technological University, Singapore City, 639798 Singapore

**Keywords:** Glioma, Meningioma, Micro-OCT, Patholgoy, Tumor discrimination

## Abstract

**Background:**

Precise identification, discrimination and assessment of central nervous system (CNS) tumors is of critical importance to brain neoplasm treatment. Due to the complexity and limited resolutions of the existing diagnostic tools, however, it is difficult to identify the tumors and their boundaries precisely in clinical practice, and thus, the conventional way of brain neoplasm treatment relies mainly on the experiences of neurosurgeons to make resection decisions in the surgery process. The purpose of this study is to explore the potential of Micro-optical coherence tomography (μOCT) as an intraoperative diagnostic imaging tool for identifying and discriminating glioma and meningioma with their microstructure imaging ex vivo, which thus may help neurosurgeons to perform precise surgery with low costs and reduced burdens.

**Methods:**

Fresh glioma and meningioma samples were resected from patients, and then slices of such samples were excised and imaged instantly ex vivo with a lab-built μOCT, which achieves a spatial resolution of ~ 2.0 μm (μm). The acquired optical coherence tomography (OCT) images were pathologically evaluated and compared to their corresponding histology for both tumor type and tumor grade discriminations in different cases.

**Results:**

By using the lab-built μOCT, both the cross-sectional and *en face* images of glioma and meningioma were acquired ex vivo. Based upon the morphology results, both the glioma and meningioma types as well as the glioma grades were assessed and discriminated. Comparisons between OCT imaging results and histology showed that typical tissue microstructures of glioma and meningioma could be clearly identified and confirmed the type and grade discriminations with satisfactory accuracy.

**Conclusions:**

μOCT could provide high-resolution three-dimensional (3D) imaging of the glioma and meningioma tissue microstructures rapidly ex vivo. μOCT imaging results could help discriminate both tumor types and grades, which illustrates the potential of μOCT as an intraoperative diagnostic imaging tool to help neurosurgeons perform their surgery precisely in tumor treatment process.

## Background

Primary brain and central nervous system (PCNS) tumors are heterogeneous groups of slow-growing benign or malignant neoplasms, which typically arise from the meninges, pituitary gland, pineal gland and nerves in the brain [1–3]. It has been reported that PCNS tumors account for 3% of all cancers, with their incidences varying among people of different ages or genders or from different countries [2–4]. Glioma and meningioma are the two most common types of PCNS tumors, and constitute more than 80% of all brain neoplasms worldwide [5, 6]. Specifically, gliomas originating from the glial cells account for more than 70% of all malignant brain tumors and are more frequently diagnosed in men than in women. While meningiomas arising from arachnoidal cells constitute more than 20% of all brain tumors and are typically benign diagnosed more frequently in women [3, 7]. Due to their unknown etiologies, however, both glioma and meningioma are difficult to be identified until severe symptoms appear [1, 3, 8, 9].

The conventional way for the treatment of glioma and meningioma is to excise the visible tumor tissues in clinical practice [2]. It has been reported that aggressive surgical resection is effective for reducing the risk of anaplastic progression in low-grade glial tumors, which thus helps prolong the survival of patients with malignant gliomas [10, 11]. However, due to the lack of reliable imaging tools to identify the interfaces between the true tumor, infiltration zone and tumor adjacent, current surgical resections rely mainly on the experiences of neurosurgeons to make the surgery decisions. Therefore, complete tumor resection is almost impossible in practice. In such a case, patients may suffer either from the inevitable recurrences if cancerous tissues are not resected completely, or from the surgery-inflicted neurological deficits with non-cancerous tissues being removed, or both [12], which would impose huge burdens on both patients and social healthcare systems. Hence, intraoperative diagnostic tools capable of providing both clear tissue structures and resection boundaries are highly desired in clinical practice.

Various imaging modalities, such as computed tomography (CT), magnetic resonance imaging (MRI), intraoperative ultrasonography (IUS), as well as positron emission tomography (PET) and Raman microspectroscopy (RM), have already been developed both for diagnosis and surgical resections of brain tumors in clinical practice [12–18]. Specifically, CT and MRI are effective as preoperative tools for their noninvasiveness and abilities to provide deep brain structure images with large field of view (FOV). However, both tools suffer from the limited spatial resolutions and imaging speed, and they are also sensitive to imaging positions and shift errors. While intraoperative CT/MRI largely addresses those issues and allows neurosurgeons to assess the resection cavity, yet it is expensive and slow, and is not suitable for real-time surgery guidance [10]. Furthermore, the radiation dose optimization problem with CT or MRI is another issue that hindering their applications as an intraoperative diagnostic imaging tool for CNS tumors surgical treatment [19–21]. In contrast, although IUS could be used for real-time imaging with large imaging depth and FOV, its spatial resolution is limited and tissue image contrast is too low to be used for clear tumor identifications [18]. PET and RM are useful for diagnostic applications owing to their subcellular-level resolutions, yet their applications are restricted by their imaging depth and FOV. Meanwhile, the radiation issue with PET and the low scattering efficiency of RM are also key factors hindering their applications. In addition, since all those existing imaging systems are complicated, they could not be integrated onto the microsurgical instruments to provide real-time images of the resection cavity in clinical practice. Hence, it is imperative to develop simple and effective intraoperative imaging modalities for brain tumor detections and discriminations for neurosurgeries.

Optical coherence tomography (OCT) is an emerging powerful imaging modality based on low-coherence interferometry and Confocal reflectance microscopy [22]. By measuring the light signals from its two interferometry arms, OCT is able to provide real-time three-dimensional (3D) images of tissue microstructures at a typical spatial resolution of 5–15 μm (μm) with an imaging depth up to 3 mm (mm) in solid tissues [23]. OCT has been utilized for various biological tissue imaging, e.g., retina, skin, gastric tract, coronary artery, and breast tissues, etc., [24–28], and was first used to acquire morphology images of animal brains ex vivo for the first time in [29]. Later, it was further utilized to acquire both cross-sectional and depth-resolved *en face* images of human brain cancer tissues ex vivo [10, 30–32]. Recently, the application of OCT has been advocated beyond structure imaging, and was adopted for detection of human brain cancer infiltration [33]. Specifically, by measuring the optical attenuation values at the cancer core, infiltrated zone and non-cancer white matter using a swept-source OCT (SS-OCT), the spatial resolution of which was 16.0 μm and 6.4 μm in lateral and axial directions, respectively, the cancerous and noncancerous tissues could be differentiated. Meanwhile, an attenuation threshold was also obtained to distinguish between cancer and noncancer white matter based on the acquired volumetric OCT images. However, although the study proved the feasibility of SS-OCT for cancer infiltration detection, no previous studies have reported on using OCT to differentiate between the two typical CNS tumors, e.g., glioma and meningioma, let alone the tumor grade classifications.

In this proof-of-concept study, we evaluate the feasibility of Micro-OCT (μOCT), which is an OCT system that achieves a spatial resolution of a few microns, as an intraoperative imaging tool for both tumor types and grade discriminations of glioma and meningioma ex vivo based on the comparisons between μOCT images and pathology. Specifically, a μOCT that achieves a spatial resolution of ~ 2.0 μm was constructed first, and then was utilized to acquire images of the freshly resected brain tumor tissues ex vivo. Results show that the typical tissue microstructures of glioma and meningioma could be clearly identified, which helps discriminate the tumor types and glioma grades. To the best of our knowledge, this is the first report on estimating spectral-domain OCT (SD-OCT) as an imaging tool for both brain tumor type identifications and grade classifications.

## Methods

### Setup of the μOCT system

**Fig. 1:**
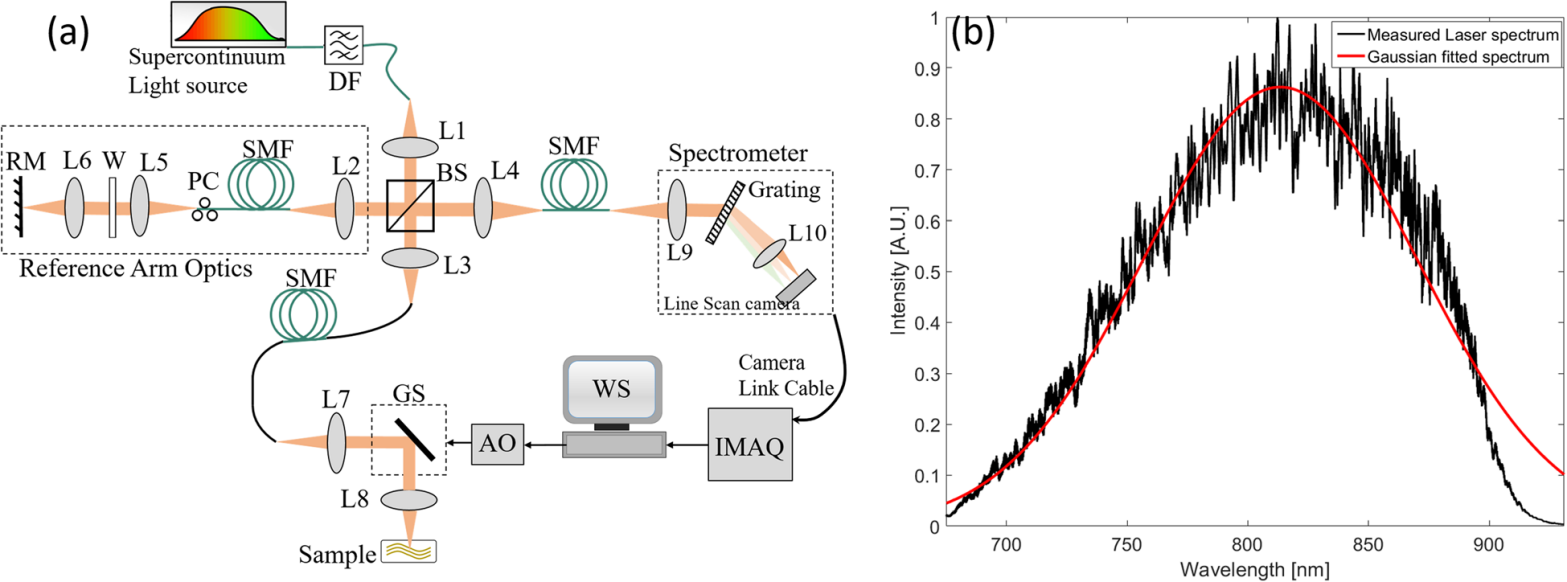
A lab-built μOCT system used for experiments. **a** The construction of the μOCT system. DF: dichroic filter; L1-L9: achromatic lens; L10: camera lens; SMF: single mode fiber; W: UV fused silica window; PC: Polarization controller; BS: beam splitter; WS: workstation; IMAQ: image acquisition board; AO: analog output; GS: galvo scanner; RM: reference mirror. **b** The laser spectrum detected by the customized spectrometer

A home-built free-space SD-OCT as shown in Fig. 1a was adopted for experiments in this study. As illustrated, a supercontinuum laser (SC-5, Yangtze Soton Laser Co., Ltd., China) with a broadband spectrum ranging from 480 nm (nm) to 2200 nm and a repetition rate of 5 Mega-Hertz (MHz) was utilized as the light source. A dichroic filter (DMSP1000, Thorlabs Inc.) with a transmission band of 520 nm to 985 nm was placed after the laser output, and thus, a spectrum centered at 810 nm with full width at half maximum (FWHM) of ~ 180 nm was achieved. Such a laser output was then coupled to the μOCT system by a collimation lens L1 (AC050–010- 115 B-ML, Thorlabs Inc., USA) and a single mode fiber (SMF) (630-HP, Thorlabs Inc., USA), and output power was measured to be 65.6 milliwatt (mW) at a tuned power ratio of 100%.

The laser output was divided into two beams by a free-space 50:50 beam splitter (BS008, Thorlabs Inc., USA), one portion of which was directed to the reference arm, while the other to the sample arm. The beam directed to the reference arm was first coupled into a SMF (630-HP, Thorlabs Inc., USA) by lens L2 (AC050–010-B-ML, Thorlabs Inc.), and then is directed to a lens pair L5 (AC050–015-B-ML, Thorlabs Inc., USA) and L6 (M Plan Apo NIR 20 × −, Edmund Optics Inc., USA), and finally was back-reflected by a reference mirror (RM). The beam directed to the sample arm optics travels exact the same components and path length as that of the reference arm, except that a galvo scanning mirror (GVSM002/M, Thorlabs Inc., USA) was inserted in between the lens pair L7 (identical to L5) and L8 (identical to L6). To compensate the dispersion caused by length difference between the SMFs within the reference and sample arm optics, a ultraviolet (UV) fused silica window W (#49–643, Edmund Optics Inc., USA) was inserted into the reference arm between L5 and L6, while a polarization controller was used to tune the polarization state such that the interference fringes between the two arm optics were maximized.

The interference signals between the sample and the reference arm optics were collected by a lens L4 (AC050–015-B-ML, Thorlabs Inc., USA), and then were sent to a customized spectrometer via a SMF (630-HP, Thorlabs Inc., USA). The spectrometer, which consists of a lens L9 (AC127–030-B-ML, Thorlabs Inc., USA), a diffraction grating (1200 l/mm @ 830 nm, Wasatch Photonics Inc., USA), a camera lens L10 (Nikon AF Nikkor 85 mm f/1.8D), and a line scan camera (E2V, AViiVA EM4), was the key component for μOCT signal detections. The collected signals were finally transferred to a workstation through camera link cables and an image acquisition card (KBN-PCE-CL4-F, Bitflow Inc., USA) at 12-bit digital resolution. The laser spectrum detected by the line scan camera was illustrated in Fig. 1b. In the experiments, both the camera and galvo scanners were synchronized by the analog output (AO) trigger generated from the workstation.

### μOCT system characterization

**Fig. 2:**
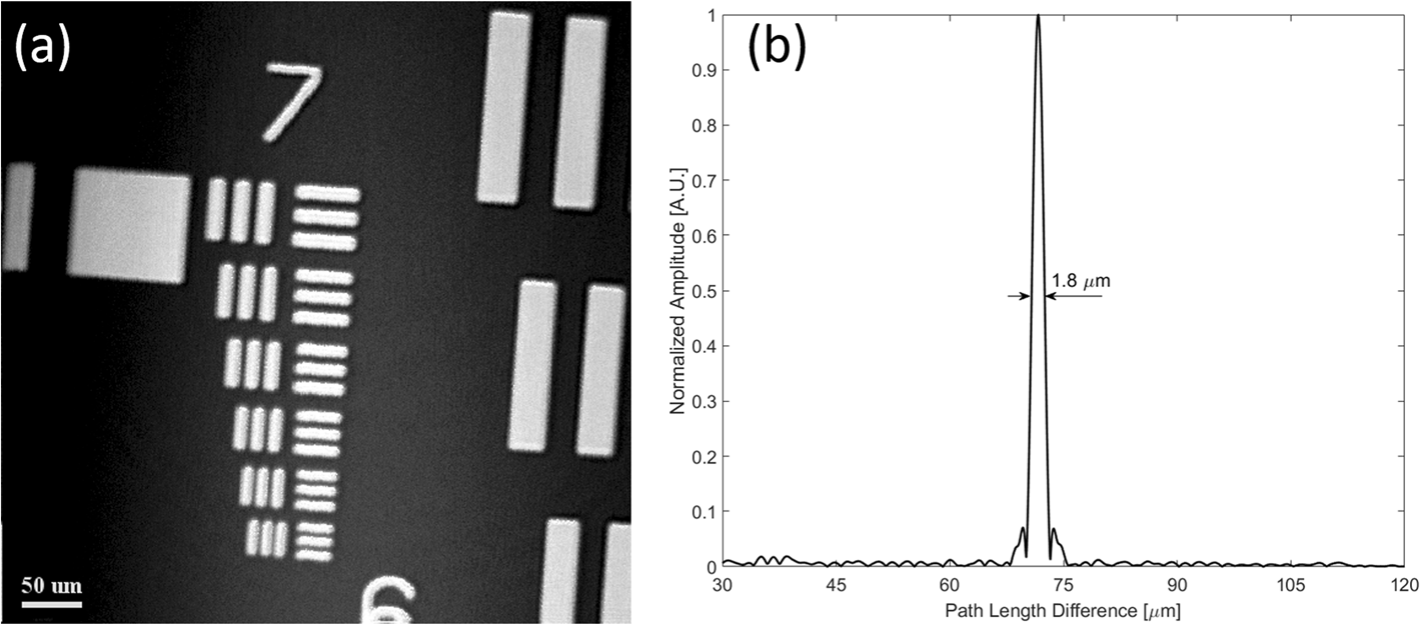
Performance characterization of the constructed μOCT system by measuring its lateral and axial resolutions. **a** *En face* image (512 pixels × 512 pixels, covering an area of 0.26 mm × 0.26 mm) of the 1951 USAF resolution chart showing the lateral resolution to be ~ 2.0 μm. **b** The measured axial PSF of a single A-line for the constructed μOCT system was ~ 1.8 μm

The μOCT system performance was characterized by its spatial resolutions. In this study, we measured the μOCT system lateral resolution by imaging a 1951 United States Air Force (USAF) resolution target to acquire its *en face* images. The obtained *en face* image shown in Fig. 2a consists of 512 pixels × 512 pixels covering an area of 0.26 mm × 0.26 mm, with the line pattern of group 7 element 6 being clearly identified. As the line spacing of such a pattern is 2.19 μm, the lateral resolution of the constructed OCT system was estimated to be ~ 2.0 μm. Such a measured value matched well with our predicted value of 2.47 μm with the fiber mode field diameter (MFD) being 4.2 μm at 810 nm.

While to characterize the μOCT system axial resolution, we inserted an actuated iris diaphragm (SM05D5, Thorlabs Inc., USA) into the sample arm optics, and then placed a BK7 prism at its focal plane. Both the reference mirror in the reference arm optics and the iris diaphragm in the sample arm optics were adjusted, such that the power for the two arms were similar at the spectrometer, meanwhile, the line scan camera was not saturated. The measured axial point spread function (PSF) of a single A-line as shown in Fig. 2b indicated that the system axial resolution was ~ 1.8 μm in air, and the signal to noise ratio (SNR) was 45.9 dB. Furthermore, since the iris diaphragm induced a power loss of 36.8 dB, while the BK7 prism caused an attenuation of 14 dB, the system sensitivity was estimated to be ~ 96.7 dB in this study.

### Study design

Freshly excised tumor specimens were utilized to conduct μOCT imaging ex vivo immediately after being extracted. This study includes 18 fresh glioma and meningioma samples that are resected from 13 patients who were diagnosed with MRI in the neurosurgery center of Renmin Hospital at Wuhan University, and both the tumor types and grades were diagnosed by neurosurgeon in the surgery process. The MRI diagnosis process, neurosurgery process, and the μOCT imaging process were operated independently. Specifically, the patients were diagnosed with MRI first, and then were arranged for neurosurgery in the hospital. Once the surgery was conducted, the tumor tissues would be collected by the surgeons, and slices of the tissues were extracted for μOCT imaging. As a preliminary work, this study is to explore the potential of μOCT as an intraoperative diagnostic imaging tool for identifying and discriminating glioma and meningioma with their microstructure imaging ex vivo. The studying protocol was reviewed and approved by the institutional review board (IRB) at Renmin Hospital of Wuhan University. (Approval Number: 2017 K-C053).

### μOCT imaging

Once the tumors were resected from patients, specimen slices would be extracted from those tumor samples and then were sent to the OCT imaging setup that was located next to the surgery room for image acquisitions. Before imaging, all excised specimens were flushed by normal saline solution to remove the blood debris on the tissue surface. While in the imaging process, a LiF glass window was fixed at the sample arm optics such that its outer surface was located at the focal plane of μOCT. Meanwhile, a small amount of ultrasound transmission gel (Aquasonic 100, Parker Laboratories Inc.) were added onto the glass window outer surface to alleviate the refractive index mismatching between the tissue and glass window surface. Each experiment with one specimen was repeated at least three times at different locations by one researcher within 3 min after the specimen being resected.

The μOCT system scanning speed was set to be 30,720 A-lines/s with each image comprised of 1024 A-lines, i.e., 30 frames/s, and thus, our μOCT system took ~ 34.1 s to acquire a volumetric image of 2.43 mm^3^, while those *en face* images were processed and evaluated offline. After imaging acquisition, the regions of interest (ROIs) been imaged were marked with tissue marking dye, and then were fixed with 10% neutral-buffered formalin for routine histological analysis.

## Results

### μOCT for meningioma and Glioma imaging

#### 3D visualization of meningioma by μOCT

**Fig. 3:**
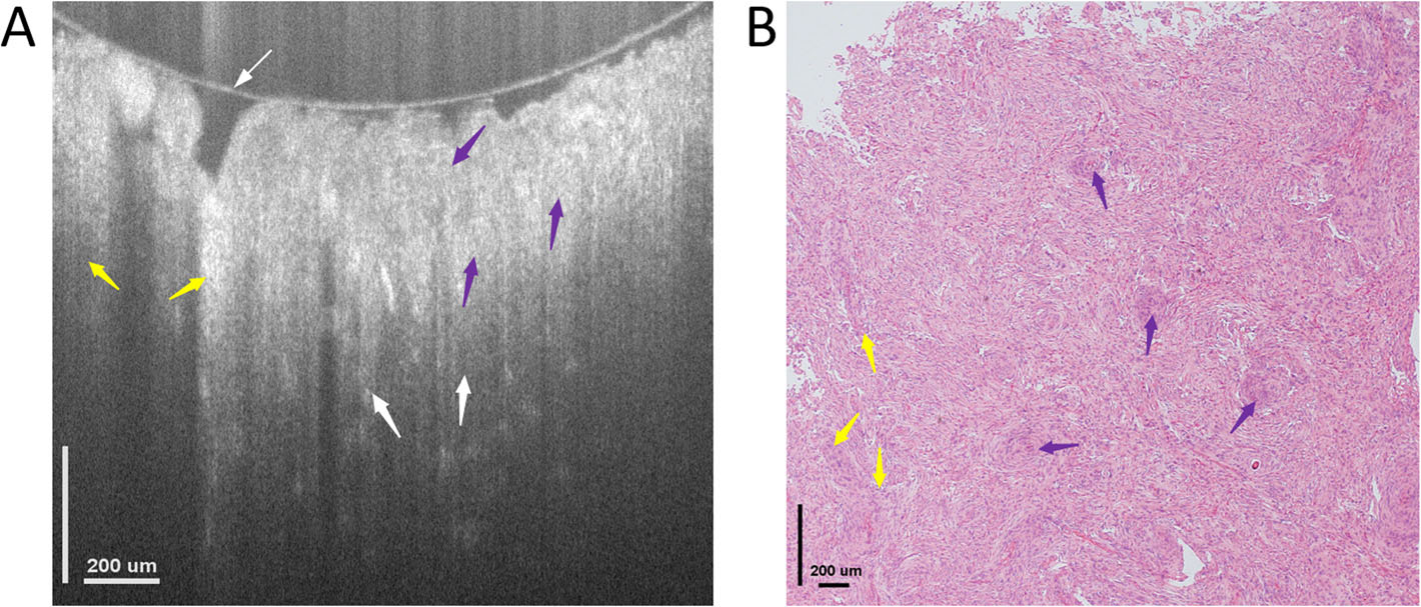
Cross-sectional image of meningioma microstructures ex vivo. **a** Cross-sectional image acquired by OCT showing the microstructures of the tissue. **b** Histology, hematoxylin and eosin: 100×. Both the Fibrous structure (yellow arrows, F) and whorl formations (purple arrows) could be identified by OCT. The thin white arrow in figure (**a**) indicates the glass surface touching the tissue surface

Figure 3a shows a typical cross-sectional μOCT image of the meningioma tissue resected from a 55-year old female patient. It consists of 924 pixels × 882 pixels covering an area of 1552 μm × 760 μm in width and depth directions, respectively. As shown in Fig. 3a, the overall tissue is comprised of dense structures without showing any typical alignments or apparent blood vessels, and some of those structures are grown in sliced shapes with the whorl formations and the fibrous microstructures distributed around randomly. Meanwhile, those detailed typical tissue microstructures that are associated with meningioma, e.g., the whorl formation and the fibrous microstructures, could be clearly identified, with those whorl formations appear to be spiral structures, while those fibrous microstructures being formulated as slices. Both of these structures are denser than the normal tissues, and they are probably introduced by the tissue cell necrosis. The dense tissue microstructures together with the whorl formations and fibrous structures are well appreciated by the OCT system with good correspondence to the histology, as shown in Fig. 3b.

**Fig. 4:**
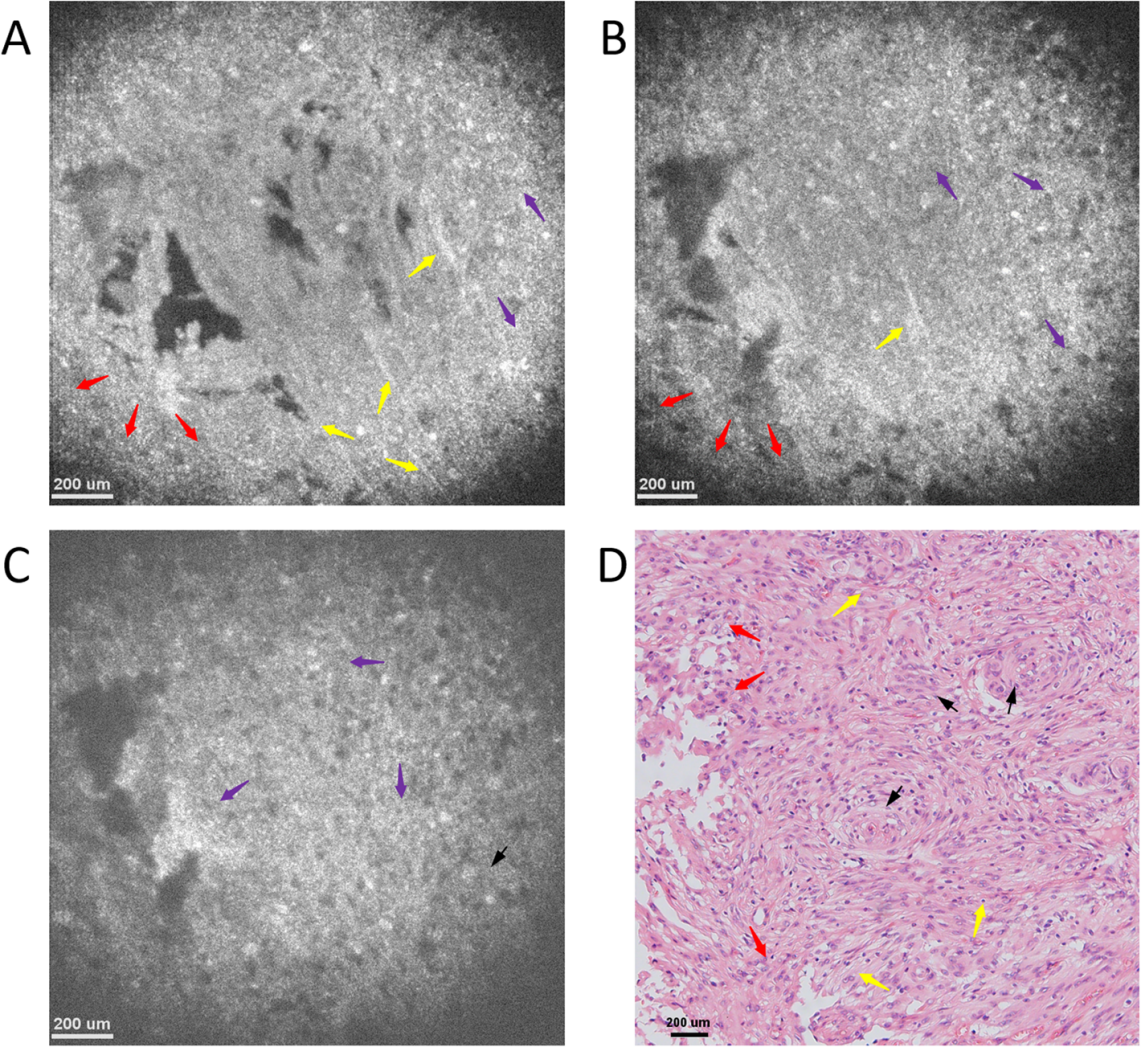
Typical OCT *en face* image of meningioma tissue at different imaging depths and their corresponding histology image. *En face* OCT image at an imaging depth of (**a**) 150 μm, (**b**) 350 μm, (**c**) 550 μm, respectively. **d** Representative *en face* histology image with hematoxylin and eosin: 100×. All OCT images consists of 886 pixels × 886 pixels, covering an area of 1.4 mm × 1.4 mm, and all typical tissue microstructures, e.g., meningothelial (red arrows), fibrous (yellow arrows) and whorl formations (purple arrows), could be identified with good correspondence to histology

The detailed structures of the samples could be further identified with the rapidly 3D reconstructed *en face* images at different imaging depths as shown in Fig. 4. Figure 4a-c present *en face* images at imaging depths of 150 μm, 350 μm and 550 μm, respectively. All those images consist of 886 pixels × 886 pixels, covering an area of 1.4 mm × 1.4 mm. As seen, the typical microstructures, e.g., meningothelial, fibrous and whorl formations, could be clearly resolved, while the characteristic nucleus cytological features, such as the uniformly distributed chromatin with empty nucleus and pseudo-inclusion in the nucleus, were also observed with good correspondence to the meningioma lesion tissue histology as shown in Fig. 4d. Both cross-sectional and *en face* images show that the overall tissue appears to be dense without showing any typical alignments, and such results are consistent with that of the histology.

#### 3D visualization of low-grade glioma by μOCT

As presented above, μOCT images could clearly illustrate the microstructures of the meningioma, and they are consistent with those in histology. Herein, we evaluate the feasibility of μOCT 3D imaging for low-grade glioma diagnosis.

**Fig. 5:**
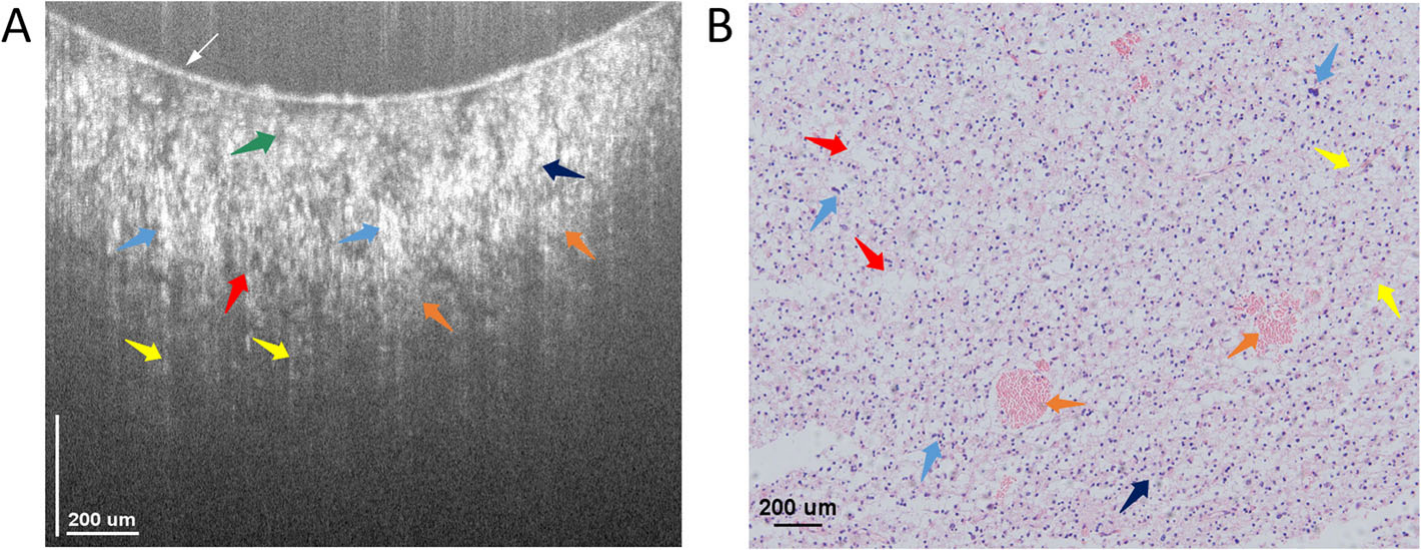
Typical OCT cross-sectional image of low-grade glioma tissue and its corresponding histology image. **a** Cross-sectional OCT image of glioma tissue. The image consists of 924 pixels × 825 pixels, covering an area of 1.5 mm × 0.7 mm in width and depth, respectively, and the typical microstructures, e.g., mucin-like stroma (green arrows), glioblastoma multiforme (blue arrows), glioma (orange arrows), as well as fibrous (yellow arrows) and vesicles (red arrows) could be identified with good correspondence to histology. **b** Typical histology image of glioma tissue image with hematoxylin and eosin: 100×

Figure 5a shows a cross-sectional OCT image of glioma ex vivo. As seen, the overall colloid structure is relatively sparse, while the nuclei density is relatively large, with the sizes of nuclei being different. The mucin-like stroma (MS) were distributed below the inner surface of glass window, while both glioblastoma multiforme (GM) and hypercellularity (HC) were resolved below MS. The GM are gathered together like massive necrosis, and the HC looks like nuclei gathering. Below such structures, the glioma (G), fibrous (F) and vesicles (V) were also resolved with good correspondence to those in the histology as shown in Fig. 5b.

**Fig. 6:**
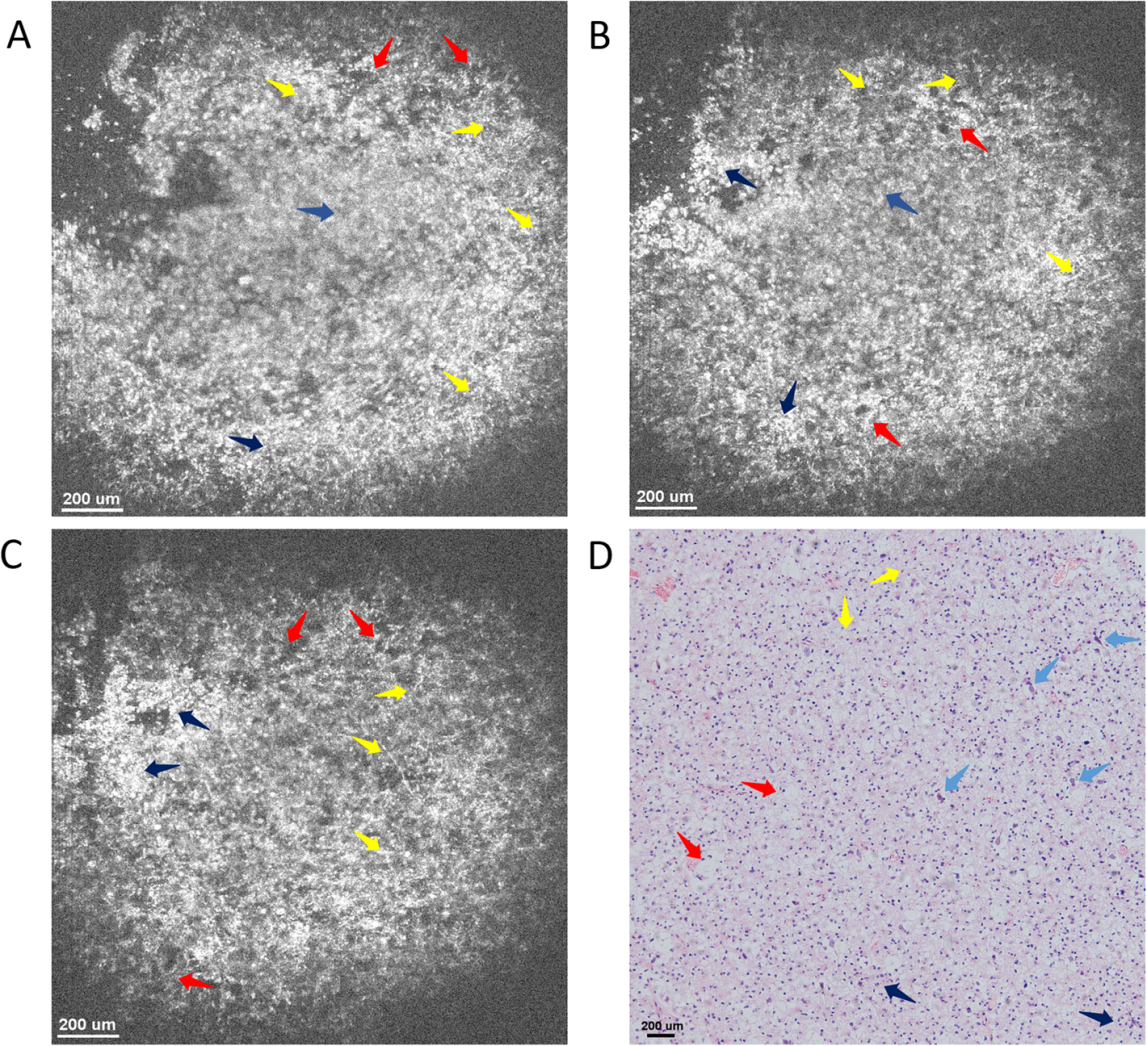
*En face* OCT images of low-grade glioma tissue ex vivo at imaging depths of (**a**) 150 μm, (**b**) 350 μm, and (**c**) 550 μm. All those images consist of 886 pixels × 886 pixels, covering an area of 1.48 mm × 1.48 mm. The typical microstructures, e.g., mucin-like stroma (green arrows), glioblastoma multiforme (blue arrows), glioma (orange arrows), as well as fibrous (yellow arrows) and vesicles (red arrows) could be clearly identified. **d** Histology of glioma tissue image with hematoxylin and eosin: 100×

Figures 6a-c present the OCT *en face* images acquired at imaging depths of 150 μm, 350 μm and 550 μm, respectively. Results confirmed that the overall tissue structure is sparse, and those mucin-like stroma and fibrous tissue were distributed uniformly in the lesion and formulated as vesicles, which thus make the tissue structure sparse due to the low reflectivity of vesicles. Furthermore, since the strip-like fibrous tissue structure distribution stays almost unchanged with an increasing imaging depth, the lesion tissue micro-structures are uniform in the vertical direction, which thus makes the glioma tissue nodular and elastic. The microstructures in both cross-sectional and *en face* images are consistent with their respective histology in Figs. 5b and 6d, demonstrating the potential of μOCT for glioma diagnosis.

#### 3D visualization of high-grade glioma by μOCT

**Fig. 7:**
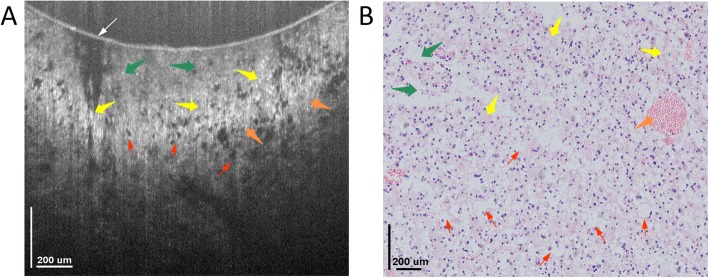
OCT cross-sectional image of high-grade glioma tissue and its corresponding histology image. **a** Cross-sectional OCT image of high-grade glioma tissue. The image consists of 924 pixels × 825 pixels, covering an area of 1.5 mm × 0.7 mm in width and depth directions, respectively. The microstructures, e.g., mucin-like stroma (green arrows), glioma (orange arrows), as well as fibrous (yellow arrows) and vesicles (red arrows) could be identified clearly. **b** Typical histology image of glioma tissue image with hematoxylin and eosin: 100×. The thin white arrow in figure (**a**) indicates the glass surface touching the tissue surface

**Fig. 8:**
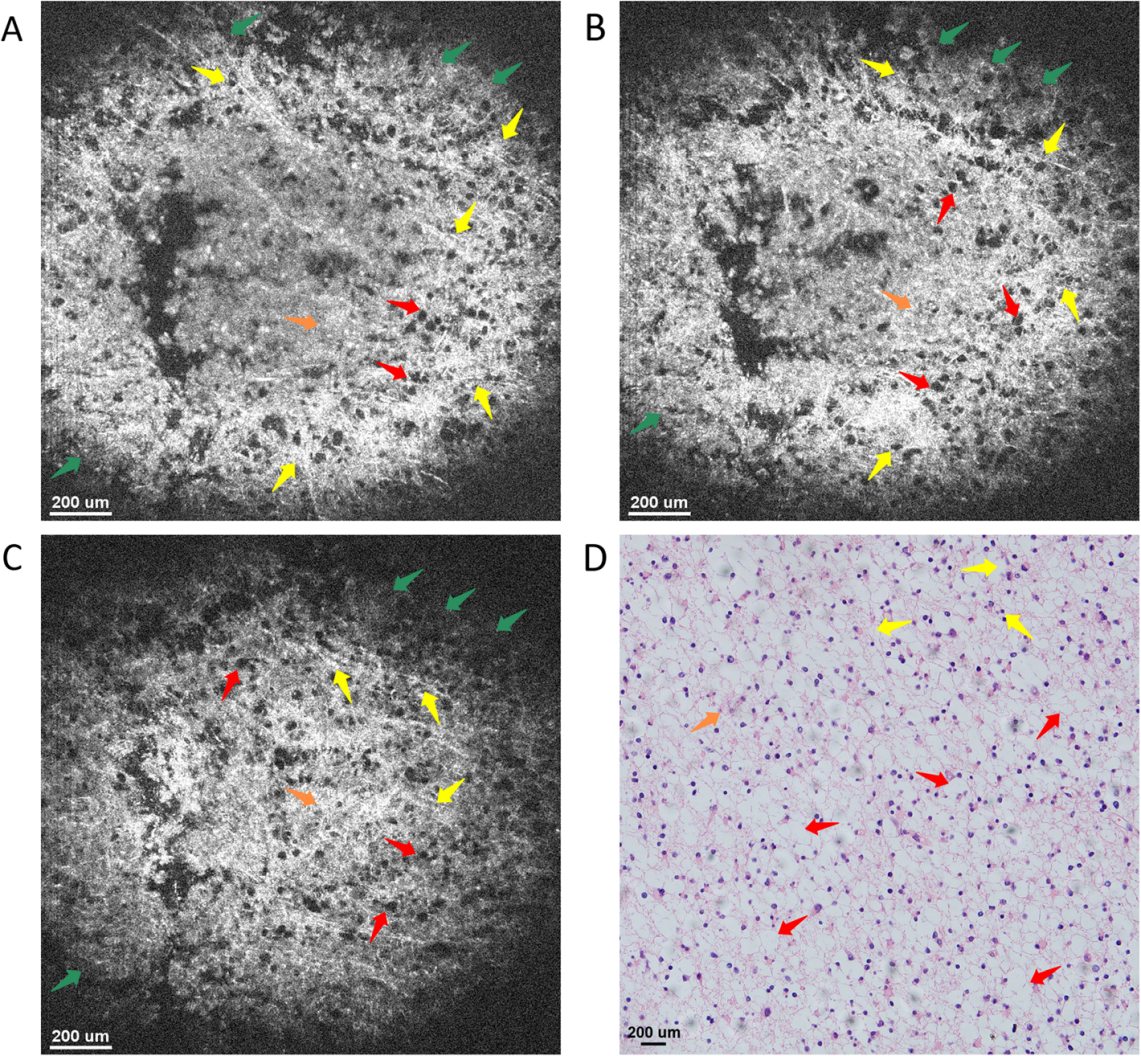
*En face* OCT images of high-grade glioma tissue ex vivo at imaging depths of (**a**) 150 μm, (**b**) 350 μm, and (**c**) 550 μm. The microstructures, e.g., mucin-like stroma (green arrows), glioblastoma multiforme (blue arrows), glioma (orange arrows), as well as fibrous (yellow arrows) and vesicles (red arrows) could be clearly identified with good correspondence to the histology. **d** Histology of glioma tissue image with hematoxylin and eosin: 100×

Figure 7a presents a cross-sectional OCT image of high-grade glioma that was diagnosed by MRI. As seen, the overall tissue microstructure is similar to that of the low-grade, yet the structural density is sparser. Specifically, the mucin-like stromata are distributed below the glass surface within the lesion tissue, while both the fibrous and gliosis structures formulated by vesicles are distributed uniformly below the mucin-like stroma. Such typical microstructures of high-grade glioma could be further verified by OCT *en face* images at different imaging depths. As shown in Figs. 8a-c, the typical microstructures, e.g., mucin-like stroma (MS, green arrows), glioblastoma multiforme (GM, blue arrows), glioma (G, orange arrows), as well as fibrous (F, yellow arrows) and vesicles (V, red arrows) could be identified clearly. The mucin-like stromata are distributed near the surface, while both fibrous structures and gliosis are distributed below. Moreover, the strip flocculent-like fibrous structures are distributed within the lesion with a high reflectivity over the FOV, yet the gliosis tissues are under the mucin-like stromata as massive shaped nuclei islands. It is also worth noting that the lesion tissue structures stay almost unchanged with an increasing imaging depth, i.e., the tissues are distributed uniformly in the depth direction. The good correspondences between OCT images and histology for both cross-sectional and *en face* images further verify the potential of μOCT as a diagnostic imaging tool for glioma diagnosis in clinical practice.

### μOCT for type and grade discriminations of meningioma and Glioma

#### Meningioma and Glioma tumor type discriminations by μOCT

The 3D morphology images were utilized for meningioma and glioma type discriminations. When comparing Figs. 5 and 6 to Figs. 3 and 4 respectively, it could be observed that significant differences exist for those different types of tumor images. Specifically, the comparison between Figs. 3 and 5 shows that the structures of Fig. 3 are relatively denser, smoother and substantial, with numerous whorl formations distributed within it. On the contrary, the structures in Fig. 5, are relatively sparser with extensive floccules distributed within it, which is formulated by the vesicles distributed among the microstructures. The comparison between Figs. 4 and 6 further demonstrate that, the meningioma structures are denser and uniform in the volumetric colloid tissues due to the distribution of the fibrous structures, while the structure of gliomas appear to be sparse, and are distributed as nodular and elastic structures due to the existence of the hypercellularity and the glioblastoma multiforme.

#### Grade discrimination for Glioma by μOCT

When comparing Figs. 5a and 7a, we could observe that the morphologies appearances in the two images are quite different even though the detailed structures are similar to each other. As shown in Fig. 5a, those tissue structures in low-grade glioma are relative denser, while Fig. 7a illustrates that, the tissue structures in high-grade glioma are sparser with numerous vesicles formulated by fibrillary and gliosis being clearly observed. The comparison between these two images also shows that the dense hypercellularity structures with high reflectivity in low-grade glioma samples were absent in the high-grade ones.

The differences between the low- and high-grade gliomas could be further verified by their respective OCT *en face* images. As shown in Fig. 6a, the tissues in low-grade glioma are relatively denser, and their distributions are almost uniform in both lateral and axial directions. While for high-grade glioma as illustrated in Fig. 8a, however, those tissue structures are relatively sparser, with numerous vesicles being observed in both axial and lateral directions. Furthermore, in the high-grade glioma, the density of the small vesicles is higher, and they are distributed in the fibrous tissues as hairy shapes, oligodendroids or multi-protuberant stars. In the low-grade gliomas, however, the fibrillary and hypercellularity are much denser distributed in clustering and striping shapes. The similar differences could also be observed at different imaging depths or different cross-sectional OCT images. The same observations could also be obtained from the extensive experiments using the other glioma lesion specimens. Such differences generate since the tissue structures are largely changed during the infiltration of high-grade glioma lesions.

## Discussions

As one of those diseases with the highest mortality, CNS tumor has attracted extensive research efforts over the past years, and various diagnostic imaging tools, e.g., US, CT, MRI as well as PET and RM, etc., have been developed for its diagnosis, which have also made significant contributions to neurosurgery. In clinical practice, however, there is always a need to differentiate the malignant glioma from the benign meningioma, or to identify the boundaries between the tumor and the normal tissue, since it determines the appropriate treatment strategies to be adopted to avoid the incomplete and/or over-resections. To fulfil such purposes, however, those modalities still suffer from certain limitations in terms of system complexity, resolution, imaging depth, FOV, etc. for precise CNS tumor diagnosis, and thus, it is extremely difficult for neurosurgeons to make rapid on-site diagnosis with the excised specimens, and they have to make resection decisions based mainly on their experiences.

In this proof-of-concept study, we explored the potential of μOCT as an intraoperative diagnostic imaging tool for meningioma and glioma types and glioma grade discriminations, for the first time to the best of our knowledge. Both cross-section and 3D imaging results show that the lab-built μOCT, which achieves a spatial resolution of ~ 2.0 μm, could provide detailed microstructures of meningioma and glioma. Imaging results of those tumor specimens demonstrated that μOCT is capable of providing real-time rapid, label-free and 3D imaging of the CNS tumors with cellular−/sub-cellular morphology information, which are comparable to those in their corresponding pathological images and could help neurosurgeons to identify both CNS tumors types and glioma grades. The noninvasiveness, high-resolution and simplicity properties make μOCT a promising candidate as an intraoperative diagnostic tool for CNS tumor diagnosis in clinical practice, while the neurosurgeons could adopt appropriate treatment strategies for the patients. Furthermore, as compared with the routine diagnostic imaging tools, the μOCT imaging-based diagnosis is not only much more cost-effective, but also much more time efficient and accurate. Typically, the routine pathological based diagnosis takes 1–3 days to experience the tedious processes, e.g., tissue excision, specimen resections, staining, and imaging, which may also introduce missed diagnosis and/or misdiagnosis. While with μOCT, the detection could be carried out instantly after tissue resection, and the diagnosis could be finished in very short time, and thereby, the neurosurgeons could make appropriate surgery decisions quickly and accurately. In addition, the μOCT system is much simpler. It has been reported that OCT, and thereby μOCT, could be miniaturized and integrated onto a handheld surgical pick tips [34, 35]. Specifically, with the high-resolution miniature OCT detection probes integrated surgical pick tips, neurosurgeons could visualize the “real-time” tissue-instrument interactions directly [36, 37], and thus, they could be able to excise the tumors completely. In such a way, the neoplasms can be treated properly, while over-resection, or missed resection, or both could be avoided. We believe that this study would lay a solid foundation for the future applications of μOCT in neurosurgery in clinical practice.

Both tissue cross-sectional and 3D morphology images were utilized for tumor types and glioma grade discriminations in this study. However, although it might be easy for pathologists to identify those tumors, the use of morphology images is still not straightforward and convincing enough, since there is no qualitative criterion for evaluation. Specifically, even though it has been reported that tissue optical attenuation values could be utilized for brain cancer infiltration identification [33], no criteria have been established for discriminating the tumor types of glioma and meningioma as well as the glioma grades. Therefore, certain criteria, especially numerical discrimination criteria are highly desired for the clinical use of μOCT. A possible parameter might be the morphology sparsity or spectroscopic contrast [38], since the tissue structures vary significantly among the glioma and meningioma as well as the glioma grades, and thus, both the sparsity and the spectroscopic contrasts could be different for those different tissue structures. However, as the number of samples is not large enough, such parameters have not been evaluated yet by the time when this manuscript was submitted. We are still collecting tissue samples to evaluate the feasibility of establishing different diagnostic criteria for discrimination purpose.

However, there still exist several limitations in this study. First, all the specimens were mainly evaluated according to their morphology appearances, and in some cases, the cell nuclei distributions, which are the typical pathological diagnosis criteria for glioma and meningioma, were not always clearly visualized in OCT images due to the limited image contrast. Furthermore, the FOV was limited to ~ 2.2 mm^3^ in this study due to the trade-off between the high spatial resolution and the large scanning area. Although such a limited imaging volume makes high-resolution real-time diagnostic possible, yet it may take much longer time to differentiate the tumor boundaries if being utilized in clinical practice. To address such issues, OCT systems with multi-scale imaging range [25] and new contrast enhancement methods [38–43], such as acetic acid stain reflectance, tissue backscattering property analysis, and artificial intelligence (AI) based image processing mechanisms, etc., could be devised and adopted for imaging. Improvements to OCT systems are also underway for acquiring more clear images of nuclei [25, 44, 45]. Second, the number of tumor samples is limited, and it is insufficient enough to measure the sensitivity and accuracy in this study. Last, accurate CNS tumor diagnosis with μOCT requires neurosurgeons to understand the OCT tumor pathology images clearly. Future studies with a larger number of samples using multi-scale imaging, random CNS tumor types and more lesion grades are to be conducted to evaluate the potential of μOCT for CNS tumor diagnosis. We believe that μOCT could be a promising candidate diagnostic tool to assist neurosurgeons make accurate clinical judgment as well as appropriate treatment strategies in the near future.

## Conclusion

In conclusion, this study presented high-resolution μOCT imaging of glioma and meningioma ex vivo, and the comparisons to their corresponding pathological images. Results convincingly demonstrated that μOCT is capable of discriminating both CNS tumor types and glioma grades, which thus illustrates the feasibility of μOCT as an intraoperative diagnostic imaging tool to help neurosurgeons perform precise surgery in tumor treatment process.

## Data Availability

The datasets generated and/or analyzed during this study are available from the corresponding author upon reasonable request.

## Abbreviations

3D: Three Dimensional
CNS: Central Nervous System
CT: Computed Tomography (CT)
F: Fibrous
FOV: Field of View
FWHM: Full Width at Half Maximum
G: Glioma
GM: Glioblastoma Multiforme
HC: Hypercellularity
IRB: Institutional Review Board
IUS: Intraoperative Ultrasonography
MFD: Mode Field Diameter
MRI: Magnetic Resonance Imaging (MRI)
MS: Mucin-like Stroma
OCT: Optical Coherence Tomography
PCNS: Primary brain and Central Nervous System
PET: Positron Emission Tomography
PSF: Point Spread Function
RM: Raman Microspectroscopy
RM: Reference Mirror
ROIs: Regions of Interest
SD-OCT: Spectral-Domain OCT
SMF: Single Mode Fiber
SNR: Signal to Noise Ratio
SS-OCT: Swept-Source OCT
UV: Ultraviolet
V: Vesicles
μOCT: Micro-Optical Coherence Tomography
